# Polymorphisms of Toll-like receptor-4 and CD14 in systemic lupus erythematosus and rheumatoid arthritis

**DOI:** 10.1186/2050-7771-1-20

**Published:** 2013-05-16

**Authors:** Tarak Dhaouadi, Imen Sfar, Youssra Haouami, Leila Abdelmoula, Sami Turki, Lamia Ben Hassine, Rafik Zouari, Adel Khedher, Narjess Khalfallah, Taieb Ben Abdallah, Yousr Gorgi

**Affiliations:** 1 Laboratory of Research in Immunology of Renal Transplantation and Immunopathology (LR03SP01) Charles Nicolle Hospital, Tunis El Manar University, Bd 9 Avril, 1006 Bab Saadoun, Tunis, Tunisia; 2 Rheumatology Department Charles Nicolle Hospital, Tunis El Manar University, Tunis, Tunisia; 3 Internal Medicine Department A Charles Nicolle Hospital, Tunis El Manar University, Tunis, Tunisia; 4 Internal Medicine Department B Charles Nicolle Hospital, Tunis El Manar University, Tunis, Tunisia

**Keywords:** Polymorphisms, TLR4, CD14, Systemic lupus erythematosus, Rheumatoid arthritis

## Abstract

**Background:**

Toll-like receptor 4 (TLR4) and its co-receptor CD14 play a major role in innate immunity by recognizing PAMPs and signal the activation of adaptive responses. These receptors can recognize endogenous ligands mainly auto-antigens. In addition, TLR4 (Asp299Gly) and CD14 (C/T -159) polymorphisms (SNPs) may modify qualitatively and/or quantitatively their expression. Therefore, they could be implied in autoimmune diseases and can influence both susceptibility and severity of systemic lupus erythematosus (SLE) and rheumatoid arthritis (RA).

**Patients and methods:**

TLR4 (Asp299Gly) and CD14 (C/T -159) SNPs were genotyped using polymerase chain reaction (PCR)-RFLP in 127 SLE patients, 100 RA patients, and 114 healthy controls matched in age and gender.

**Results:**

CD14*T allele was significantly more frequent in SLE patients (0.456) comparatively to controls (0.355), p = 0.02 OR (95% CI) = 1.53 [1.04-2.24]. In RA patients, the higher frequency of CD14*T allele (0.405) failed to reach significance, p = 0.28. Investigation of the TLR4 (Asp299Gly) SNP showed no significant association neither with SLE nor with RA.

Analysis of these SNPs according to clinical and biological features showed a significant higher frequency of arthritis in SLE patients carrying CD14*T/T genotype (92%) comparatively to those with C/C and C/T genotypes (72.5%), p = 0.04. Moreover, SLE patients carrying CD14*T/T/TLR4*A/A haplotype had significantly more arthritis (91.3%) than the rest of SLE group (73%), p = 0,044 and confirmed by multivariable analysis after adjustment according to age and gender, p = 0.01.

**Conclusion:**

The CD14 (-159)*T allele seems to be associated with susceptibility to SLE and arthritis occurrence.

## Introduction

Systemic lupus erythematosus (SLE) and rheumatoid arthritis (RA) are common complex diseases characterized by a chronic generalized inflammation that may involve several tissues and organs. Currently, SLE and RA are pigeonholed in *non-specific-organ* autoimmune diseases because of the abundance of immune complexes, presence of autoantibodies, genetic association with human leucocyte antigen specificities and build-up of immune system cells (lymphocytes, macrophages, neutrophils) within pathological lesions.

In spite of significant advances in understanding of pathogenic mechanisms of these diseases, physiopathological puzzles are still incomplete. Presently, defects in cell-apoptosis are known to be involved in initiation and sustainability of both SLE and RA. In fact, during SLE there is an increased apoptotic load due to accelerated apoptosis of keratinocytes by UV-rays and decreased clearance by phagocytes that leads to redistribution, modification and persistence of auto-antigens (basically nuclear) which may activate auto-reactive T and B cells [1]. All through RA, again there is an apoptosis defect of *fibroblast-like synoviocytes* (FLS) due to an increased expression *in situ* of an anti-apoptotic molecule FLIP (Fas-associated death domain Like Interleukin-1 converting enzyme Inhibitory Protein) [2,3]. These immortal FLSs invade synovia and cartilage and load out metalloproteases leading to their destruction and release of extracellular matrix debris (EMD).

These nuclear auto-antigens (SLE) and EMD (RA) are *damage associated molecular patterns* (DAMPs) that might be recognized by Toll-like receptors (TLRs), mainly TLR4 and its co-receptor CD14 paving the way to secretion of pro-inflammatory cytokines (IL-6 and TNFα). In fact, inappropriate TLR4 activation induces chronic inflammation and, therefore autoimmune complications [4].

TLR4 and CD14 genes exhibit many polymorphisms (SNPs) that might influence qualitatively and/or quantitatively their expression. The Asp299Gly SNP in TLR4 gene which was functionally associated to significant decrease of response to LPS [5,6] could play a protective role against autoimmune disorders such as SLE and RA. Inversely, the C/T -159 SNP in the gene promoter of CD14 enhances its transcriptional activity and increases levels of both membrane (mCD14) and soluble (sCD14) expression that might lead to raised inflammation and potentially be implied in predisposition to SLE and RA [7].

In this context, both TLR4 (Asp299Gly) and CD14 (C/T-159) SNPs were investigated in Tunisian patients with SLE or RA in order to look for a potential impact on susceptibility and/or disease severity.

## Materials and methods

### Patients

This study included 127 patients with SLE, 100 patients with RA and 114 healthy voluntary blood donors from the same geographic origin. Patients were visiting both internal medicine departments (A and B) and rheumatology department of the Charles Nicolle Hospital in Tunis and were diagnosed according to the Criteria Committee of the American College of Rheumatology [8,9]. Clinical and biological features of patients are recorded in Table 1.

**Table 1:** Clinical and biological features of SLE and RA patients

**SLE patients**	**n = 127**	**RA patients**	**n = 100**
Mean Age ± DS (years)	32,48 ± 13,59	Mean age ± DS (years)	40,43 ± 14,48
Median of evolution ± DS (years)	5 ± 4,96	Median of evolution ± DS (months)	120 ± 97,46
Sex ratio (Female/Male)	6,47 (110/17)	Sex ratio (Female/Male)	3,54 (78/22)
		Early onset before 40 years	51 (51%)
Cutaneous lesions n (%)	77 (60,6%)	Bone erosions n (%)	38 (38%)
Arthritis n (%)	97 (76,3%)	Subcutaneous nodules n (%)	17 (17%)
Nephritis n (%)	68 (53,5%)		
Titer of ANA > 1/200 n (%)	106 (83,4%)	Rheumatoid factor (RF) n (%)	75 (75%)
Anti-dsDNA antibodies (Abs) n (%)	90 (70,8%)	Anti-CCP antibodies (ACCPA) n (%)	73 (73%)
Decreased complement activity (DCA) n (%)	93 (73,2%)	HLA-DRB1*04 allele	46 (46%)
		Shared epitope	46 (46%)
SLEDAI* ≥ 8 n (%)	91 (71,6%)	Active RA (DAS28† ≥ 5,1) n (%)	70 (70%)
		DAS28† (average ± DS)	5,97 ± 1,72

* SLE disease activity index: a score meant to measure the activity in SLE in patients.

† Disease activity score 28: a score for evaluation of RA activity by assessing the state of 28 joints.

Controls were healthy subjects matched for age, gender and ethnicity. None of the healthy subjects had any evidence of personal or family history of autoimmune disease.

All patients and controls gave written informed consent to participate in the study, and the local Ethics committee of Charles Nicolle Hospital approved this study.

### Methods

Genomic DNA was extracted from peripheral blood using salting-out procedure [10]. The identification of TLR4 (Asp299Gly) and CD14 (C/T -159) SNPs was performed by *polymerase chain reaction* (PCR) using specific primers [(TLR4*F: 5′-GAT TAG CAT ACT TAG ACT ACT ACC TCC ATG -3′ and TLR4*R: 5′-GAT CAA CTT CTG AAA AAG CAT TCC CAC-3′) and (CD14*F: 5′- GTG CCA ACA GAT GAG GTT CAC-3′ and CD14*R: 5′-GCC TCT GAC AGT TTA TGT AAT C-3′) (metabion^®^ international AG, Lena-Christ-strasse 44I, D-82152 Martinsried, Deutshland)], followed by a digestion of amplification products using *NcoI* and *AvaII* respectively (*Restriction fragment length polymorphism*: RFLP).

### Statistical analysis

Univariable analysis was performed using chi-square test or fisher’s exact test for small numbers (Epi-info Stat 6.04 program CDC, Atlanta). Probability (*p*) values were corrected for the number of tested alleles (pc). Values < 0.05 were considered to be statistically significant. Frequencies of genotypes and alleles were analyzed by chi-square test. In order to evaluate the strength of associations, the odds ratios (OR) together with 95% confidence intervals (CI) were calculated. Logistic regression models were built according to age, gender to estimate adjusted ORs. Statistical powers were calculated in order to evaluate the strength of used tests.

## Results

### TLR4 Asp299Gly analyses

The results of TLR4 genotyping in SLE and RA patients and controls are summarized in Table 2. We observed no significant differences in genotypes and alleles frequencies between patients (SLE or RA) and controls.

**Table 2:** Results of TLR4 Asp299Gly and CD14 (C/T -159) SNPs genotyping in patients and controls

**Polymorphism TLR4 Asp299Gly**	**Controls n = 114**	**SLE n = 127**	***p***	**OR (95% CI)**	**RA n = 100**	***p***	**OR (95% CI)**
**Genotypes**							
**A/A**	102 (89.4%)	111 (87.4%)	0.61	0.82 [0.34-1.93]	82 (82%)	0.11	0.54 [0.23-1.25]
**A/G**	12 (10.5%)	16 (12.5%)			18 (18%)		
**Alleles**							
**A**	0.947	0.937	0.62	0.83 [0.36-1.89]	0.91	0.13	0.56 [0.25-1.27]
**G**	0.052	0.062			0.09		
**CD14 C/T -159**							
**Genotype**							
**CC**	45 (39.4%)	36 (28.3%)			32 (32%)		
**CT**	57 (50%)	66 (51.9%)			55 (55%)		
**TT**	12 (10.5%)	**25 (19.6%)**	0.048*	**2.08 [0.94-4.67]**	13 (13%)	0.57	1.27 [0.41-3.16]
**Allele**							
**C**	0.644	0.543			0.594		
**T**	0.355	**0.456**	**0.02†**	**1.53 [1.04-2.24]**	0.405	0.28	1,24 [0.82-1.86]

* *p *comparing the CD14*T/T genotype prevalence between SLE patients and controls.

† *p *comparing the CD14*T allele frequencies between SLE patients and controls.

Statistical powers: TLR4 genotypes in SLE = 12.2%; TLR4 genotypes in RA = 47.3%; CD14 genotypes in SLE = 62.6%; CD14 alleles in SLE = 47.9% and CD14 genotypes in RA = 14.2%.

In SLE patients analysis of TLR4 SNP along with clinical (cutaneous lesions, arthritis and lupus nephritis) and biological features of SLE showed no significant correlations between this polymorphism frequencies and clinical manifestations, or with increased disease activity (SLEDAI > 8), or with an elevated titer of anti-nuclear antibodies (ANA), the presence of anti-dsDNA (double stranded DNA) antibodies (Abs) and decreased complement activity (DCA).

In RA patients, again, there were no significant differences between genotypes frequencies and the age of onset, the disease activity, the presence of bone erosions, the occurrence of subcutaneous nodules, the positivity of serological markers [rheumatoid factor (RF) and/or anti-cyclic citrullinated peptide (ACCPA)] and the association of genetic factors (HLA-DRB1*04 allele or shared epitope).

### CD14 C/T -159 analyses

We found that CD14*T/T genotype and CD14*T allele were significantly more frequent in patients with SLE (19.6% and 0.456) comparatively to controls (10.5% and 0.355); *p* = 0.048, OR (95% CI) = 2.08 [0.94-4.67]; p = 0.02, OR (95% CI) = 1.53 [1.04-2.24], respectively (Table 2). Examination of CD14 SNP according to clinical and biological features of SLE showed a significant association of the homozygous mutated genotype CD14*T/T with the occurrence of arthritis; *p* = 0.04, OR (95% CI) = 4.35 [0.9-28.6] (Table 3).

**Table 3:** Arthritis in SLE patients frequencies according to CD14 C/T -159 genotypes

**CD14 C/T -159**	**CC n = 36**	**CT n = 66**	TT* n = 25	***p***	**OR (95% CI)**
**Arthritis n = 97**	**24 (66,6%)**	**50 (75,5%)**	**23 (92%)**	**0,04**	**4,35 [0,9-28,6]**

* *p *comparing arthritis frequencies between patients carrying CD14*TT genotype and those with other genotypes.

This higher frequency of both homozygous genotype CD14*T/T and CD14*T allele was also observed in RA patients but the difference failed to reach the threshold of significance (Table 2). Study of CD14 genotypes distribution alongside RA revealed no relationships with clinical and biological items. However, ACCPA were more frequently positive in patients carrying homozygous genotype CD14*T/T (84.6%) comparatively to those with *C/C and *C/T genotypes (71.2%) but the difference was not statistically significant.

### TLR4/CD14 genotypes associations

Investigation of TLR4/CD14 genotypes associations showed a significant correlation of TLR4*AA/CD14*TT with SLE (*p* = 0.035) but not with RA. Moreover, TLR4*AA/CD14*TT was significantly associated with arthritis occurrence in SLE patients (*p* = 0.01 after adjustment with age of onset and gender).

## Discussion

Toll-like receptor 4 and its co-receptor CD14 recognize both pathogen associated molecular patterns (PAMPs) and DAMPs and signal the activation of adaptive responses through enhancing expression of cytokines and co-stimulation molecules. Repeated injections of low-dose LPS (ligand of TLR4/CD14) to female MRL/n, BXSB, or NZW mice accelerates the development of lupus-like syndrome, increasing the production of autoantibodies and worsening the renal function impairment [11]. A recent study performed on transgenic mice for gp96 (a LPS chaperone that amplifies the TLR4-dependent response) showed that, on this genetic background, the commensal flora spontaneously caused the production of anti-dsDNA antibodies and the development of glomerulonephritis, via TLR4 activation [12]. The exact mechanism responsible for the development of autoimmunity remains unknown. Several hypotheses have been made. First, TLR4 ligands induce the production of pro-inflammatory cytokines such as IL-6, which are known to be involved in the pathophysiology of SLE and RA. Second, under some conditions, TLR4 ligands can induce the production of large amounts of type 1 interferon (IFN). Finally, TLR4 activation acts synergistically with other ligands, most notably those activating endosomal TLRs (TLR7 and TLR9), to stimulate the dendritic cells (DCs). The impact of TLR4 inactivation has been investigated in C57BL/6lpr/lpr mice and lupus severity was markedly reduced. More specifically, decreases were observed in autoantibody titers, renal disease severity, and levels of IFNα and IL-6 [4].

Currently, only few studies were interested in investigation of TLR4 and/or CD14 SNPs impact on SLE and RA, and to our knowledge our study is the first to publish results in a Tunisian population. The TLR4 Asp299Gly SNP results in a substitution of an aspartate by a glycine in the codon 299 which involves the extracellular domain of the receptor and may reduce its aptitude to bind several ligands [6], thus playing a protective role against inflammatory responses.

In this study, TLR4 Asp299Gly SNP was not correlated neither to susceptibility to SLE, nor to its clinical presentation, nor to disease biological features. These results corroborate those published in literature. In fact, in a study performed on 122 Spanish SLE patients and 199 controls, genotypes and alleles frequencies were quite similar [13]. Moreover, Kang et al. [14] found that the mutated allele TLR4*G was absent in both 31 Korean patients and 80 controls, thus confirming the extreme scarcity of this allele in Asian populations [15].

Also, we found no association of this SNP with RA predisposition or severity and with the positivity of serological markers. This absence of correlation with RA susceptibility and/or severity was equally observed in the majority of studies [13,16-18] all but the one performed on a Dutch population [19] including 282 patients and 314 controls, in which the homozygous genotype TLR4*A/A was significantly associated to RA occurrence; *p* = 0.025; with an estimated relative risk (RR) of 1.7.

The low frequency of the TLR4*G, and the almost absence of the homozygous mutated genotype TLR4*G/G in several populations [13,16-18]; including our; may hide a potential protective role against inflammatory and autoimmune responses like those observed in SLE and RA. Indeed, TLR4*G allele was associated to an increased risk of infections [20], and mainly to *Respiratory Syncytial Virus* infections in infants [21] and to pneumococcal [22] and *Candida Albicans* infections [23]. In addition, this mutation had been associated to occurrence of severe sepsis [24] through important secretion of important amounts of pro-inflammatory cytokines (TNFα and IL-6). Those morbidities associated to TLR4 Asp299Gly SNP may cause an increase of mortality and lead to the observed low frequencies of the TLR4*G allele and the almost absence of the TLR4*G/G genotype that could theoretically protect against SLE and RA.

The functional variant CD14*T that enhances the promoter activity and increases the CD14 expression was markedly associated to SLE susceptibility in the present study but was not correlated to both disease activity and severity. This was also the case in a study performed in an Indian population [25] including 100 patients and 112 controls in which the mutated allele CD14*T was significantly associated to SLE with an OR (95% CI) of 2.76 [1.32-5.79]; *p* = 0,008. Unfortunately, in this study the authors had not detailed the impact of this SNP on disease severity and outcome. These data confirm the important role of CD14 which levels have been formerly identified as a predisposition factor to SLE [26] and correlated to disease activity and its prognosis [27] suggesting its use for disease monitoring. In addition, corticosteroid therapy that improves clinically and biologically SLE patients had been associated to a significant decrease of sCD14 [28]. Along with all these arguments, CD14 C/T -159 functional SNP seems to play a major role in susceptibility to SLE, nevertheless it is necessary to confirm this result on independent cohorts and with family studies.

Even, lacking significance the CD14*T allele was similarly more prevalent in patients with RA compared to controls. Additionally, homozygous genotype CD14*T/T was non-significantly correlated to the presence of ACCPA. Only 2 studies had investigated the role of this polymorphism in RA and both concluded that the CD14 C/T -159 SNP do not constitute a predisposition factor to RA and do not influence its severity [29,30]. Inversely, levels of sCD14 were correlated to RA susceptibility, disease activity and occurrence of radiological damage [29,30] confirming that CD14 is deeply involved in RA pathogenesis. The fact that the investigation of CD14 C/T -159 SNP failed to confirm its impact might be due to, first genetic and epigenetic factors that could influence the gene transcription, second to a weak role that could be missed in case control studies. Therefore, larger sample of patients is required to establish a final conclusion as to CD14 C/T-159 SNP real effect on both susceptibility and severity of RA.

Therefore, in order to provide a strong confirmation of the association of CD14 SNP with the SLE susceptibility and the non-significant correlation with RA, functional study of gene expression by CD14 mRNA assessing and sCD14 measuring in sera is needed.

## Conclusion

In Tunisian, CD14-159*T allele seems to be associated to SLE susceptibility but not to its severity.

## Competing interests

No benefits in any form have been received or will be received from a commercial party related directly or indirectly to the subject of this article.

## Authors’ contribution

Pr GY proposed the study and wrote the first draft. TD analyzed the data. All authors contributed to the design and interpretation of the study and to further drafts. Pr GY is the guarantor of the integrity of this study. All authors read and approved the final manuscript.
